# Perioperative outcomes and learning curves of minimally invasive hysterectomy: a comparative analysis of MPLH, RASPH, and SPLH

**DOI:** 10.3389/fmed.2026.1888457

**Published:** 2026-07-15

**Authors:** Ying Zhang, Xiaofeng Xu, Wenyou Zhu, Yao Liu, Qin Shi, Fuyun Dong, Rujun Chen

**Affiliations:** 1 Department of Gynecology and Obstetrics, Jiading District Central Hospital Affiliated Shanghai University of Medicine & Health Sciences, Shanghai, China; 2 Department of Gynecology and Obstetrics, Shanghai Fifth People’s Hospital, Fudan University, Shanghai, China; 3 Center of Community-Based Health Research, Fudan University, Shanghai, China

**Keywords:** learning curve, minimally invasive hysterectomy, perioperative outcomes, robot-assisted surgery, single-port laparoscopy

## Abstract

**Objective:**

To compare perioperative safety, efficiency and learning curves of MPLH, RASPH and SPLH, and analyze surgical disparities stratified by uterine volume. We adopted multivariable regression (primary adjusted analysis) and multinomial IPTW weighting (sensitivity analysis) to correct indication confounding; though IPTW failed to balance baseline covariates and could not achieve adequate covariate balance, both adjusted methods yielded consistent outcomes.

**Methods:**

This single-center retrospective cohort enrolled 280 patients (118 MPLH, 35 RASPH, 127 SPLH). We compared intraoperative metrics, early recovery indicators, 30-days complications and CUSUM learning curves, stratifying patients into simple (<200 cm^3^) and complex (≥200 cm^3^) uterine subgroups. Raw comparisons were supplemented by two covariate-adjusted models controlling for uterine volume, BMI, pelvic adhesions and benign/malignant pathology.

**Results:**

Unadjusted operative time differed markedly (Kruskal–Wallis *P* < 0.001): MPLH 83.9 [80.7, 125.0] min, RASPH 166.0 [137.0, 170.0] min, SPLH 102.0 [88.5, 107.0] min. Robotic console time excluded docking steps. After adjustment, RASPH independently required longer operating time, with comparable efficiency between MPLH and SPLH. Unadjusted blood loss varied significantly (*P* < 0.001): MPLH 126 [115, 140] mL, RASPH 134 [93.5, 143] mL, SPLH 156 [146, 170] mL. Raw blood loss was similar between MPLH and RASPH, yet SPLH lost more blood; adjustment confirmed equivalent hemorrhage for MPLH and RASPH. Hospital stay and POD1 pain scores were comparable (all *P* > 0.05), and 30-days complication rates were balanced (3.4% vs. 5.7% vs. 3.1%, *P* = 0.79). RASPH took longer in both uterine subgroups, but its complex subgroup only contained seven patients with limited statistical power. CUSUM inflection points were 15 (MPLH), 19 (SPLH) and 23 (RASPH); only 12 robotic cases fell after the threshold, weakening plateau reliability.

**Conclusion:**

The three techniques shared similar short-term safety. Adjusted data identified prolonged operative time for RASPH, while MPLH and SPLH had equivalent efficiency. SPLH was associated with greater intraoperative bleeding, whereas blood loss was comparable between MPLH and RASPH. Severe baseline imbalance restricts causal subgroup interpretation. MPLH and SPLH reached proficiency faster, offering evidence for personalized surgery and tiered training; prospective balanced cohorts are needed for further validation.

## 1 Introduction

Hysterectomy is a common surgical procedure for the treatment of benign and malignant gynecologic diseases, and minimally invasive approaches have gradually replaced open surgery as the gold standard due to their advantages of less trauma, faster recovery and lower complication rates (1, 2). Multiport laparoscopic total hysterectomy (MPLH), robot-assisted single-port total hysterectomy (RASPH) and single-port laparoscopic total hysterectomy (SPLH) are three widely used minimally invasive hysterectomy techniques in clinical practice, each with unique technical characteristics and clinical application scenarios (3–7). MPLH is a classic laparoscopic approach with mature technical specifications, while RASPH is praised for its three-dimensional vision and flexible operative instruments, and SPLH represents the development direction of minimally invasive surgery with the advantage of better cosmetic outcomes and reduced port-related complications.

Despite the widespread application of these three techniques, there remains a lack of direct head-to-head comparative studies on their perioperative outcomes, especially regarding differences in operative efficiency and learning curve characteristics. Moreover, uterine volume, a key clinical factor affecting surgical difficulty, may lead to heterogeneous operative outcomes of different techniques in subgroups of simple and complex cases, which has not been fully elucidated in previous research. Clarifying the perioperative efficacy and safety of MPLH, RASPH and SPLH, as well as their subgroup performance based on uterine volume and learning curve differences, is crucial for gynecologic surgeons to select individualized surgical approaches and optimize clinical training strategies.

This study aimed to conduct a retrospective comparative analysis of the three minimally invasive hysterectomy techniques, focusing on perioperative metrics, subgroup differences in operative time based on uterine volume, and learning curve inflection points. We hypothesized that SPLH would exhibit comparable safety and superior operative efficiency to MPLH and RASPH, with more significant advantages in simple cases with small uterine volume, and that minimally invasive techniques with simpler operational processes would have a steeper learning curve. The findings of this study are expected to provide evidence-based clinical references for the selection and promotion of minimally invasive hysterectomy techniques.

## 2 Materials and methods

### 2.1 Study design and population

A single-center retrospective cohort study was conducted on patients who underwent minimally invasive hysterectomy (MPLH, RASPH or SPLH) at the Department of Gynecology and Obstetrics, Jiading District Central Hospital Affiliated Shanghai University of Medicine & Health Sciences, between January 2023 and April 2026. The inclusion criteria were: (1) pathological diagnosis of benign or early malignant gynecologic diseases requiring hysterectomy; (2) no prior history of abdominal or pelvic malignant tumor surgery; (3) complete perioperative clinical and follow-up data; (4) uterine volume measurable by preoperative transvaginal ultrasound or MRI. Supplementary clarification: The early malignant gynecologic diseases included herein refer only to low-risk early cervical cancer and early endometrial cancer without extrauterine metastasis; all these early malignant cases receive standardized minimally invasive hysterectomy within our department’s routine clinical workflow. The exclusion criteria were: (1) severe cardiopulmonary, hepatic or renal dysfunction incompatible with minimally invasive surgery; (2) pelvic adhesion grade III–IV; (3) conversion to open surgery for non-technical reasons; (4) incomplete clinical data.

A total of 280 eligible patients were enrolled in the study, with 118 patients in the MPLH group, 35 in the RASPH group, and 127 in the SPLH group. The study was approved by the Institutional Review Board of the Jiading District Central Hospital Affiliated Shanghai University of Medicine & Health Sciences (No. 2024K02). The IRB granted a full waiver of written informed consent for this retrospective chart-review research. All clinical data used in this analysis were fully de-identified before extraction, no direct patient contact was required, and retrospective collection of signed consent would be logistically impracticable while only posing minimal risks to patient privacy. All procedures were performed in strict accordance with the ethical principles outlined in the Declaration of Helsinki.

### 2.2 Surgical techniques

**FIGURE 1:**
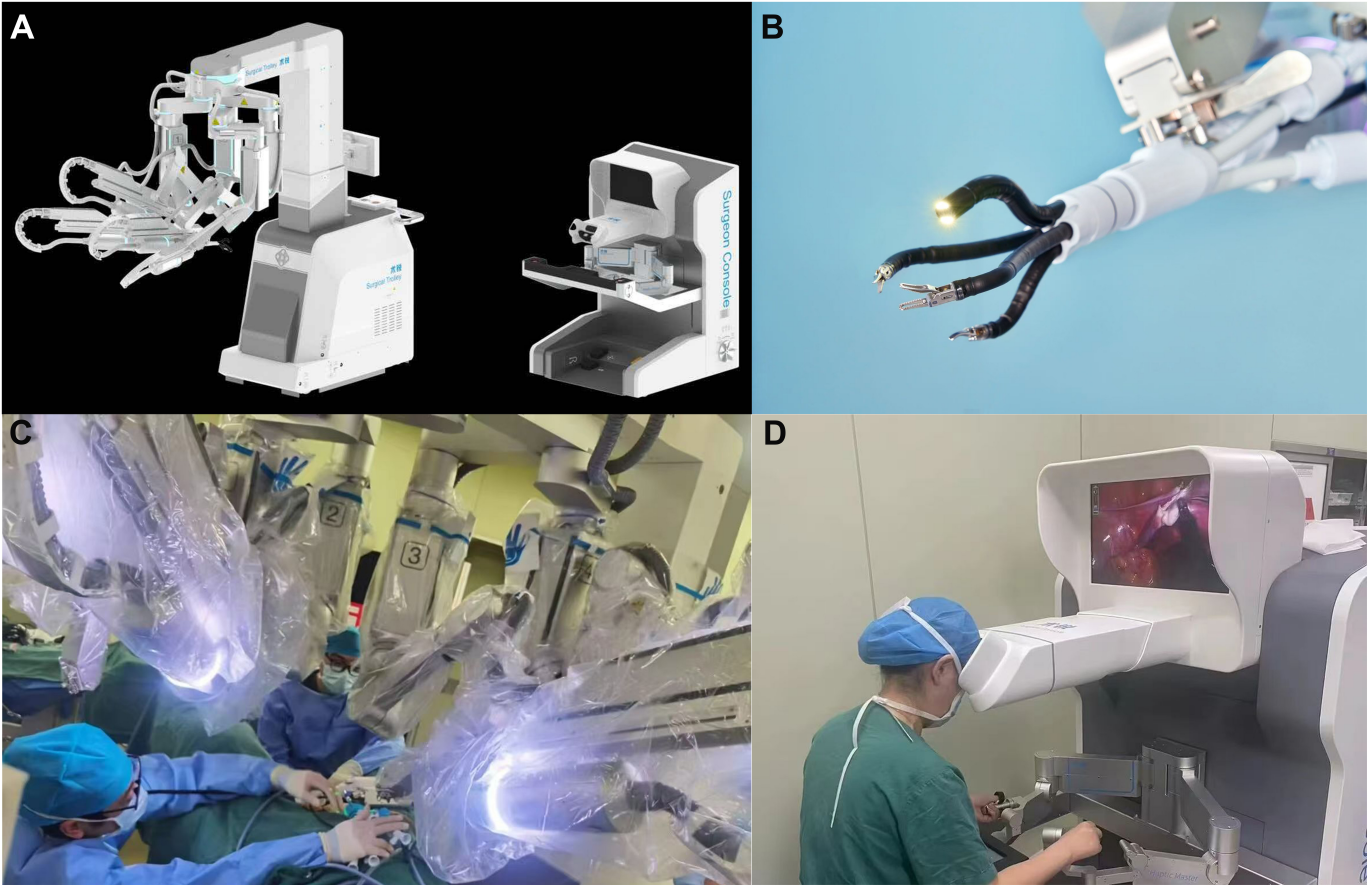
The SHURUI single-port robotic surgical system and its intraoperative application. **(A)** The SHURUI^®^ single-port robotic surgical system (Model SR-ENS-600; Beijing Surgerii Robotics Co., Ltd., Beijing, China), showing the patient-side surgical trolley with articulated robotic arms and the surgeon console. **(B)** Close-up of the high-flexibility serpentine surgical instruments (diameter: ∼5 mm) with 7 degrees of freedom, enabling multi-angle bending of the tip for precise manipulation in the narrow pelvic cavity. **(C)** Intraoperative setup of the robotic arms in a total hysterectomy procedure, with sterile drapes applied to the instruments and patient. **(D)** The surgeon operating at the console, using the integrated 3D high-definition stereoscopic vision system to perform tissue dissection and suturing in real time.

Surgeries were completed by members of our gynecologic team with different levels of surgical experience. Surgeons performing MPLH were new to laparoscopic hysterectomy, while those performing SPLH and RASPH were experienced in multiport laparoscopy but newly started with single-port and robotic surgery. All operative steps of the three techniques were fully standardized. The surgical procedures for each technique were standardized as follows:

(1) MPLH: Performed via a 4–5 port laparoscopic approach, with standard hysterectomy procedures including uterine artery ligation, parametrial dissection and vaginal cuff closure.
(2) RASPH: Conducted using the domestically developed SHURUI^®^ single-port robotic surgical system (Model SR-ENS-600; Beijing Surgerii Robotics Co., Ltd., Beijing, China). This system features a trauma-reducing single-port access device integrating three instrument channels and one optical channel (Figure 1A). It is equipped with high-flexibility serpentine instruments (≈5 mm diameter) with seven degrees of freedom, enabling >±90° tip bending for precise pelvic dissection and minimizing the “chopstick effect” via a triangulation strategy (Figure 1B). The integrated 3D high-definition endoscope supports real-time fluorescence imaging to enhance anatomical visualization and dissection precision (Figure 1C). Intraoperative setup and console-based manipulation were standardized (Figure 1D). All key steps, including uterine artery ligation, parametrial dissection, and vaginal cuff closure, followed the same surgical principles as MPLH and were completed using robotic instruments (Supplementary Video 1).
(3) SPLH: Performed via a single umbilical port using a multi-channel single-port laparoscopic device, with all surgical instruments inserted through the single port to complete the hysterectomy procedure.

Notably, all surgeons performing SPLH and RASPH already possessed mature multiport laparoscopic hysterectomy experience before starting single-port/robotic cases; these cumulative sum (CUSUM) curves only reflect the learning phase of novel single-port/robotic platforms for laparoscopically experienced operators, and do not represent the full learning trajectory for trainees with zero laparoscopic background.

### 2.3 Outcome measures

#### 2.3.1 Primary outcome measures

(1) Operative time: For MPLH and SPLH, operative time was defined as the total duration from skin incision to skin suture and recorded in minutes. For RASPH, only pure console operating time was collected, excluding robotic system setup, docking and arm adjustment procedures. Notably, all robotic pre-operative preparation steps were entirely excluded from the recorded operative endpoint, so the longer procedural time observed in the RASPH cohort cannot be attributed to docking or system setup workflows.
(2) Learning curve inflection point: Determined via sequential cumulative sum (CUSUM) analysis of consecutive case operative time. The modality-specific target stable operative time was defined as the median operating duration of all post-inflection patients for each technique separately. Cumulative deviation for each sequential case k was calculated as the sum of the difference between each individual case’s operative time and the group-specific stable target time. The inflection point was defined as the first case where the CUSUM curve transitioned from a steep descending slope to a near-horizontal plateau, signifying stable surgical proficiency. Two independent blinded researchers visually identified inflection points with consensus to reduce subjective bias. Bootstrap resampling (1,000 replicates per group) was performed to generate 95% confidence intervals for each inflection threshold, with the 2.5th and 97.5th percentiles of bootstrap inflection values defining CI bounds.

#### 2.3.2 Secondary outcome measures

(1) Intraoperative blood loss: Measured by the volume of suctioned blood plus the weight of blood-soaked gauze, recorded in milliliters.
(2) Postoperative hospital stay: Calculated as the number of days from surgery to discharge, with discharge criteria including stable vital signs, no abdominal pain, normal bowel function and ability to ambulate independently.
(3) Postoperative Day 1 (POD1) VAS pain score: Assessed using a 10-point visual analog scale (VAS; 0 = no pain, 10 = worst imaginable pain). All patients across three groups received identical standardized multimodal perioperative analgesia regimens to eliminate confounding variation in pain control that could alter VAS scores. Pain assessment was limited to a single POD1 timepoint due to incomplete serial pain documentation in electronic medical records; repeated pain measurements on other postoperative days were not uniformly available for retrospective analysis.
(4) Postoperative complication rate: The proportion of patients with postoperative complications including infection, bleeding, urinary tract injury and intestinal obstruction within 30 days after surgery.

#### 2.3.3 Subgroup analysis

Patients were stratified into simple (<200 cm^3^) and complex (≥200 cm^3^) subgroups according to preoperative uterine volume quantified via transvaginal ultrasound or pelvic MRI. Uterine volume was calculated using the standard prolate ellipsoid formula: Uterine volume (cm^3^) = 0.523 × length × width × anteroposterior diameter, where the constant 0.523 approximates π/6. Three orthogonal maximal uterine dimensions were measured on standardized imaging sequences. All volumetric assessments were independently conducted by two blinded senior radiologists to reduce inter-observer variability; any inconsistent measurements were reconciled through joint review. Subgroup-specific comparisons of operative time were performed to evaluate technique-dependent surgical performance across distinct uterine size thresholds.

### 2.4 Statistical analysis

All statistical analyses were performed using R (version 4.5.1). Categorical data were reported as counts and percentages. The Shapiro–Wilk test was used to assess normality of all continuous variables, and testing revealed that operative time, intraoperative blood loss, postoperative hospital stay, Day 1 VAS pain score, age and BMI all deviated significantly from a normal distribution (all *P* < 0.05). Therefore, all unadjusted raw continuous data are uniformly presented as median (interquartile range, IQR); mean ± standard deviation was not used to describe crude observational data. For non-normal continuous outcomes, overall intergroup differences were assessed via the Kruskal–Wallis H test, followed by Bonferroni-corrected Wilcoxon rank-sum pairwise comparisons, while Fisher’s exact test was adopted for categorical variables. Standardized nonparametric effect size was calculated as absolute *Z*-value divided by the square root of total sample size (*N* = 280), with magnitude thresholds defined as negligible (r < 0.1), small (0.1 ≤ r < 0.3), medium (0.3 ≤ r < 0.5), large (r ≥ 0.5); all *r*-values are summarized in Supplementary Table 3. Cumulative sum (CUSUM) analysis was used to identify the learning curve inflection point for each technique, with full CUSUM calculation rules, target time definition, inflection adjudication and bootstrap 95% CI derivation detailed in Section “2.3.1 Primary outcome measures.” We conducted a subgroup analysis stratified by a uterine volume cutoff of 200 cm^3^ (<200 cm^3^ for simple cases, ≥200 cm^3^ for complex cases) to observe operative time differences across varying uterine sizes; however, this single threshold could not sufficiently resolve the prominent baseline imbalance in uterine volume across groups, which introduced significant confounding by indication. To better isolate the independent effect of surgical type on operative time and intraoperative blood loss, we implemented two separate parametric adjusted analytical pipelines distinct from unadjusted nonparametric rank-based tests: (1) Multivariable linear regression, where operative time and intraoperative blood loss were set as separate dependent variables, with covariates including preoperative uterine volume, BMI, pelvic adhesion status (yes/no), and pathological diagnosis (benign versus early malignant); regression coefficients and corresponding 95% CIs were calculated to quantify the independent association of each surgical approach after covariate adjustment, and full regression outputs are listed in Supplementary Table 2, with model-derived adjusted marginal means interpreted separately from unadjusted median/IQR crude data. (2) Multinomial propensity-score inverse probability of treatment weighting (IPTW): a multinomial logistic model incorporating identical four confounders was built to calculate propensity scores for each patient, individual weights were defined as the reciprocal of the predicted probability of receiving the actual surgery each patient underwent, and extreme weights were trimmed between 0.01 and 0.99 to minimize bias. Standardized mean differences (SMD) were used to evaluate covariate balance before and after weighting (SMD < 0.1 = acceptable balance). After IPTW trimming, SMD values of uterine volume, BMI, pelvic adhesion and tumor malignancy all remained far above 0.1, meaning this weighting strategy failed to eliminate severe baseline imbalance across groups. To further test the robustness of our findings against inadequate covariate balance, we additionally conducted entropy balancing as a sensitivity analysis; this alternative weighting method also failed to achieve SMD < 0.1 for uterine volume and BMI. Propensity score matching was not adopted as the primary analysis because it would exclude a large proportion of the limited RASPH cohort (total *n* = 35) and further reduce statistical power, especially for large uterus subgroup comparisons with only seven robotic patients. Dual analytical pipelines including multivariable linear regression and IPTW-weighted regression yielded consistent results to partially mitigate bias interpretation concerns. Weighted linear regression with Bonferroni-corrected pairwise tests was then performed for operative time comparisons, and covariate balance statistics alongside weighted parametric results are summarized in Supplementary Table 3. All statistical tests were two-sided, and *P* < 0.05 was considered statistically significant.

## 3 Results

### 3.1 Baseline characteristics

A total of 280 patients who underwent minimally invasive total hysterectomy were retrospectively included, with 118 in the MPLH group, 35 in the RASPH group, and 127 in the SPLH group. Age and body mass index (BMI) were comparable across all groups (all *P* > 0.05). However, preoperative uterine volume differed significantly across groups, with median [IQR] values of 278 [202, 361] cm^3^ for MPLH, 115 [78, 150] cm^3^ for RASPH, and 118 [81, 154] cm^3^ (*P* < 0.001). The distributions of comorbidities and surgical indications were generally balanced, except for adenomyosis and uterine leiomyomas, reflecting real-world clinical characteristics (Table 1). Among all participants, only four patients had early malignant gynecologic lesions without extrauterine metastasis: three cases of early endometrial cancer in the RASPH group (8.6% of RASPH patients) and one case of early cervical cancer in the SPLH group (0.8% of SPLH patients), while no malignant cases were enrolled in the MPLH cohort. Malignant cases were highly unevenly distributed across surgical groups, which may act as a confounding factor for unadjusted comparisons of operative metrics; therefore, pathological status (benign versus early malignant) was incorporated as a covariate in multivariable regression and IPTW analyses to mitigate this bias. All operations were performed by four attending surgeons within the same departmental team with fixed surgical assignments: two surgeons completed all 118 MPLH cases, two surgeons performed all 127 SPLH cases, and all 35 RASPH procedures were carried out by a single dedicated surgeon. Case volumes per individual surgeon were unbalanced, and the exclusive single-surgeon allocation for robotic hysterectomy creates inherent confounding between surgical technique and surgeon-specific experience, operative habits and procedural speed. Standardized operative steps were mandated for all three approaches to reduce inter-operator variability, yet individual surgeon identity could not be added as an extra covariate in regression and IPTW models due to the small RASPH sample size, which would lead to model overfitting. There was a nearly 2.5-fold difference in median uterine volume between the MPLH group and the other two cohorts, indicating severe baseline imbalance that would confound crude comparisons of operative efficiency. Beyond uterine size, pelvic adhesion and tumor pathological status also exhibited notable intergroup imbalance, as quantified by standardized mean differences in Supplementary Table 3. Only stratification by a single 200 cm^3^ uterine threshold could not adequately resolve this multi-dimensional selection bias; adjusted regression and IPTW analyses were therefore conducted to account for this disparity.

**TABLE 1:** Clinical baseline characteristics and perioperative outcomes stratified by surgical modality.

Variable	MPLH (*n* = 118)	RASPH (*n* = 35)	SPLH (*n* = 127)	Omnibus *P*-value
Demographic and preoperative parameters				
Age (years), median [IQR]	51.0 [48.0, 56.0]	50.0 [47.0, 54.0]	51.0 [47.0, 55.0]	0.762
BMI (kg/m^2^), median [IQR]	23.8 [21.6, 26.1]	22.9 [21.3, 24.6]	22.8 [21.2, 24.3]	0.356
Preoperative uterine volume (cm^3^), median [IQR]	278 [202, 361]	115 [78, 150]	118 [81, 154]	<0.001
Intraoperative outcomes				
Operative time (min), median [IQR]	83.9 [80.7, 125.0]	166.0 [137.0, 170.0]	102.0 [88.5, 107.0]	<0.001
Intraoperative blood loss (mL), median [IQR]	126 [115, 140]	134 [93.5, 143]	156 [146, 171]	<0.001
Postoperative outcomes				
Postoperative hospital stay (days), median [IQR]	3.0 [3.0, 4.0]	3.0 [3.0, 3.0]	3.0 [3.0, 4.0]	0.549
Postoperative day 1 pain score, median [IQR]	4.8 [4.2, 5.3]	4.4 [4.1, 4.8]	4.5 [4.0, 5.1]	0.537
Total hospital cost (Chinese Yuan), median [IQR]	20720 [19640, 22150]	29610 [28430, 30960]	23470 [22510, 24820]	<0.001
Comorbidities, *n* (%)				0.418
Anemia	5 (4.2)	2 (5.7)	3 (2.4)	
Asthma	0 (0.0)	1 (2.9)	0 (0.0)	
Breast cancer history	3 (2.5)	0 (0.0)	1 (0.8)	
Diabetes mellitus	5 (4.2)	0 (0.0)	11 (8.7)	
Fatty liver disease	12 (10.2)	1 (2.9)	7 (5.5)	
Hepatic cyst	0 (0.0)	2 (5.7)	0 (0.0)	
Hypertension	13 (11.0)	4 (11.4)	11 (8.7)	
Hyperthyroidism	0 (0.0)	1 (2.9)	0 (0.0)	
Surgical indications, *n* (%)				<0.001
Adenomyosis	58 (49.2)	4 (11.4)	42 (33.1)	
Early cervical cancer	0 (0.0)	0 (0.0)	1 (0.8)	
Early endometrial cancer	0 (0.0)	3 (8.6)	0 (0.0)	
HSIL	6 (5.1)	3 (8.6)	1 (0.8)	
Uterine leiomyomas	54 (45.7)	20 (57.1)	74 (58.2)	
Uterine prolapse	0 (0.0)	5 (14.3)	9 (7.1)	
30-days postoperative complications, *n* (%)				0.825
Bladder injury	1 (0.8)	0 (0.0)	0 (0.0)	
Intestinal injury	0 (0.0)	1 (2.9)	0 (0.0)	
Urinary retention	3 (2.5)	1 (2.9)	4 (3.1)	

All continuous variables failed Shapiro-Wilk normality testing (all P < 0.05) and are presented as median [interquartile range, IQR]. Between-group comparisons for continuous outcomes were performed using Kruskal-Wallis omnibus tests with Bonferroni-corrected post hoc pairwise comparisons; the adjusted pairwise P-values are listed below: operative time (MPLH vs. RASPH *P* < 0.001, MPLH vs. SPLH *P* = 0.634, RASPH vs. SPLH *P* < 0.001), intraoperative blood loss (MPLH vs. RASPH *P* = 1.00, MPLH vs. SPLH P < 0.001, RASPH vs. SPLH *P* < 0.001), preoperative uterine volume (MPLH vs. RASPH *P* < 0.001, MPLH vs. SPLH *P* < 0.001, RASPH vs. SPLH *P* = 0.947), and total hospital cost (MPLH vs. RASPH *P* < 0.001, MPLH vs. SPLH *P* < 0.001, RASPH vs. SPLH *P* < 0.001). Categorical variables are reported as *n* (%). Global intergroup differences for each categorical domain (comorbidities, surgical indications, 30-days postoperative complications) were evaluated via Fisher’s exact test, and the corresponding omnibus P-value is shown in the final column of the table. All participants received unified standardized multimodal perioperative analgesia protocols to reduce confounding bias in postoperative Day 1 VAS pain assessments.

### 3.2 Overall perioperative outcomes

#### 3.2.1 Primary outcomes

**FIGURE 2:**
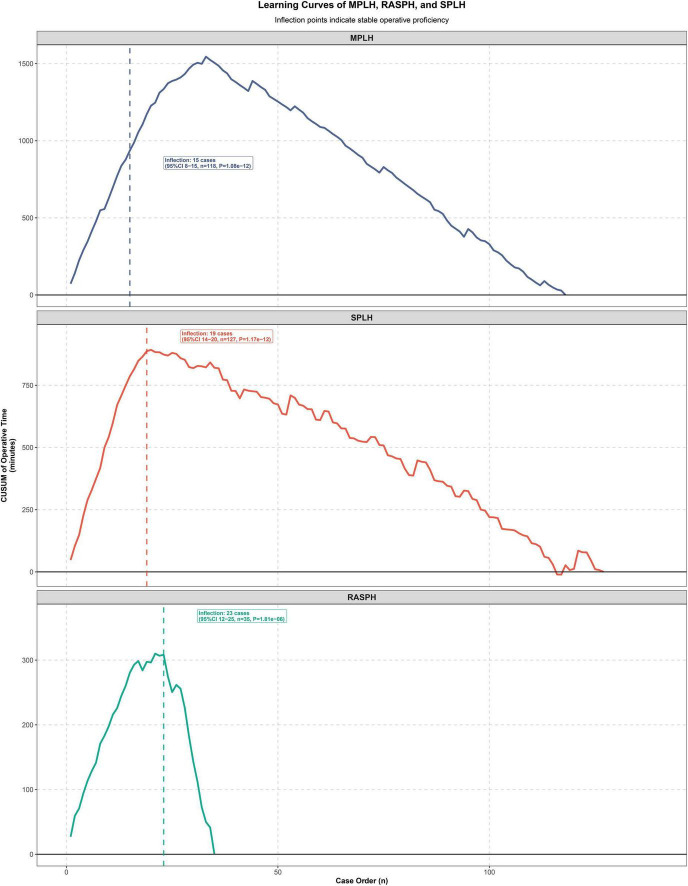
Cumulative sum (CUSUM) learning curves of MPLH (*n* = 118), SPLH (*n* = 127) and RASPH (*n* = 35). The RASPH curve inflects at case 23, with only 12 post-inflection cases available to estimate the stable operative plateau, limiting statistical precision of the robotic proficiency benchmark.

Crude intergroup comparisons revealed significant distributional differences in unadjusted operative time across the three cohorts via Kruskal–Wallis omnibus test (*P* < 0.001). Median [IQR] operative time was 83.9 [80.7, 125.0] min for MPLH, 166.0 [137.0, 170.0] min for RASPH, and 102.0 [88.5, 107.0] min for SPLH. It should be noted that recording criteria differed between robotic and laparoscopic groups, which needs to be considered when interpreting raw temporal differences. Bonferroni-corrected Wilcoxon rank-sum pairwise comparisons confirmed the operative time distribution was significantly longer for RASPH relative to both MPLH and SPLH (both adjusted *P* < 0.001, large standardized effect sizes r > 0.5), while the distribution difference between MPLH and SPLH was non-significant (adjusted *P* = 0.63, negligible effect). Critically, the recorded duration for RASPH exclusively reflected pure console operating time, with all robotic docking, system setup and arm positioning steps excluded from this metric; the prolonged procedural time observed in RASPH cannot be attributed to pre-operative robotic preparation workflows. This intra-cavity time disadvantage was likely driven by instrument crowding within the narrow single-port channel and limited cumulative robotic surgical experience among our surgical team, rather than robotic system preparation. CUSUM learning curve analysis identified proficiency inflection points at 15 cases for MPLH, 19 cases for SPLH and 23 cases for RASPH (Figure 2). The robotic cohort only contained 35 sequential cases with merely 12 patients recorded after the inflection threshold, so this robotic proficiency breakpoint should be interpreted cautiously.

Given severe imbalance across multiple core baseline covariates that introduced prominent indication bias, two parametric adjusted analytical approaches were performed to isolate the independent impact of surgical modality on operative time, distinct from the unadjusted nonparametric rank comparisons above. Multivariable linear regression adjusted for uterine volume, BMI, pelvic adhesions and pathological status showed that RASPH remained independently associated with prolonged operative duration. Relative to MPLH, the model-estimated adjusted marginal mean operative time was 61.75 min longer for RASPH (β = 61.75, 95% CI 47.57–75.92, *P* < 0.001). No meaningful independent difference was detected between SPLH and MPLH (β = 7.14, 95% CI −5.45 to 19.73, *P* = 0.27). Uterine volume, BMI and adhesion status did not exert significant independent effects on operative time after full covariate adjustment (all *P* > 0.05). Complete regression results are provided in Supplementary Table 2.

Multinomial propensity-score IPTW weighting further validated these adjusted model findings. After weight trimming between 0.01 and 0.99, standardized mean differences for all four adjusted covariates (uterine volume, BMI, pelvic adhesion and tumor malignancy) remained far above the 0.1 balance threshold with almost no improvement in baseline imbalance, indicating severe unresolvable intergroup confounding even after propensity-score adjustment. Weighted linear regression pairwise comparisons confirmed RASPH required significantly longer operative time than MPLH (mean difference = −53.40, *P* < 0.0001) and SPLH (mean difference = 49.78, *P* < 0.0001), while the weighted operative time gap between MPLH and SPLH remained non-significant (*P* = 1.00). Covariate balance statistics and weighted operative time data are presented in Supplementary Table 3.

#### 3.2.2 Secondary outcomes

Crude intraoperative blood loss distributions differed significantly across the three surgical cohorts (Kruskal–Wallis *P* < 0.001). Median [IQR] blood loss was 126 [115, 140] mL for MPLH, 134 [93.5, 143] mL for RASPH, and 156 [146, 170] mL for SPLH. Bonferroni-corrected Wilcoxon rank-sum pairwise tests demonstrated no significant crude difference in blood loss between MPLH and RASPH (*P* = 1.00), while hemorrhage was significantly greater in SPLH versus both MPLH and RASPH (both *P* < 0.001, medium standardized effect sizes 0.32–0.37). Multivariable linear regression adjusted for uterine volume, BMI, pelvic adhesions and pathological status revealed no independent difference in blood loss between RASPH and MPLH (β = −3.43, *P* = 0.544). By contrast, SPLH was independently associated with higher intraoperative hemorrhage relative to MPLH (β = 32.42, *P* < 0.001). Uterine volume, BMI, pelvic adhesion and malignant pathology exerted no independent significant effects on intraoperative blood loss (all *P* > 0.05). Full regression coefficients, 95% CIs and adjusted marginal blood loss values are summarized in Supplementary Table 2. Postoperative hospital stay and Day 1 VAS pain scores were similar across all three groups (all *P* > 0.05). The overall 30-days complication rate was low and balanced (MPLH: 3.4%; RASPH: 5.7%; SPLH: 3.1%, *P* = 0.79). Single bladder injury occurred in the MPLH group and one intestinal injury was recorded in the RASPH group; urinary retention rates were comparable among cohorts (Table 1).

### 3.3 Subgroup analysis of operative time

**FIGURE 3:**
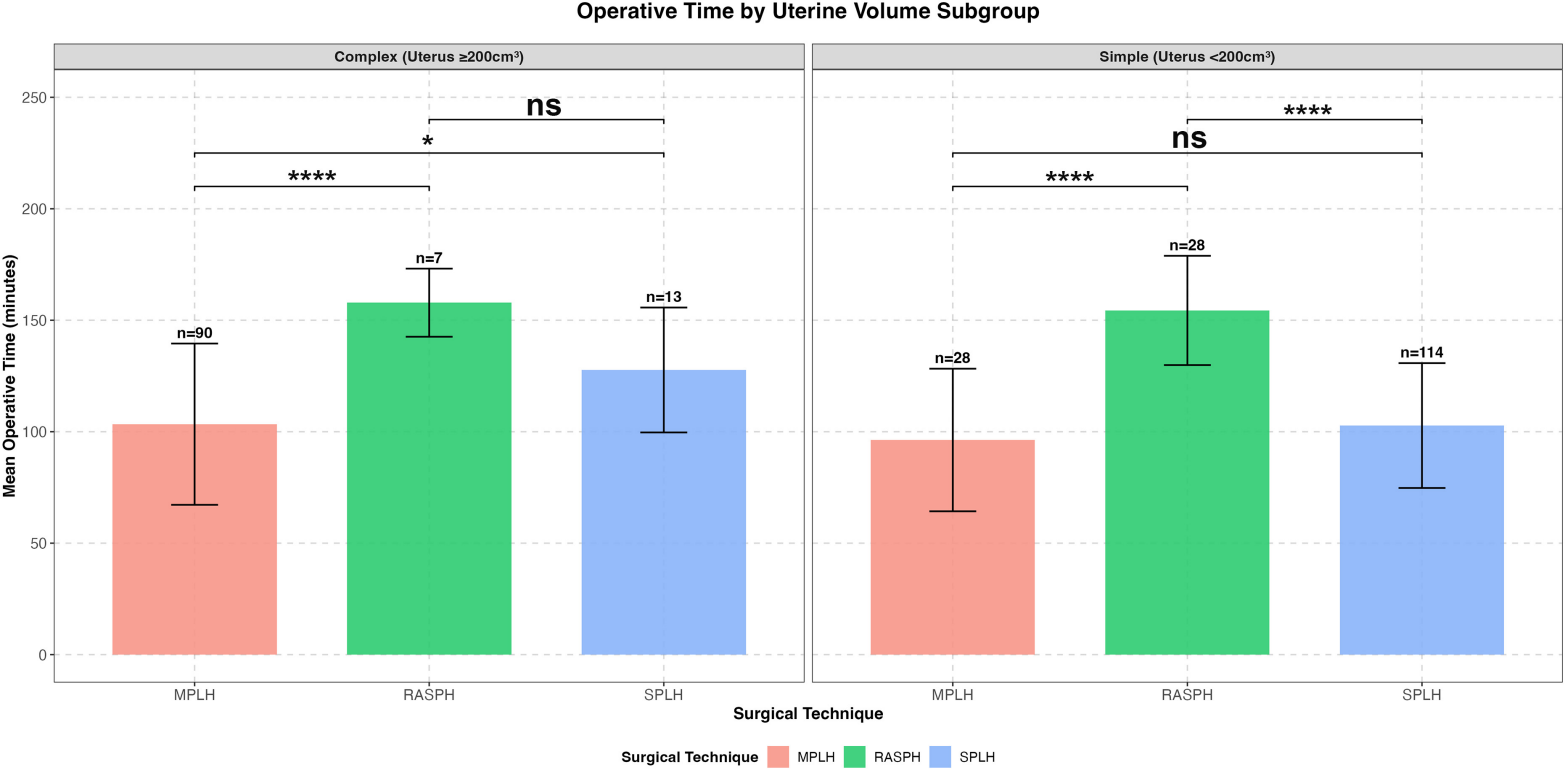
Adjusted mean operative time of total hysterectomy stratified by surgical modality and uterine volume subgroup. Sample sizes for each subgroup: MPLH (simple uteri, *n* = 28; complex uteri, *n* = 90); RASPH (simple uteri, *n* = 28; complex uteri, *n* = 7); SPLH (simple uteri, *n* = 114; complex uteri, *n* = 13). All pairwise comparisons within each stratum were adjusted using the Bonferroni correction. Comparisons involving the RASPH complex uterine subgroup are interpreted as exploratory only, given the extremely small sample size (*n* = 7) and highly unbalanced cell counts across the three modalities. *P < 0.05, ****P < 0.0001.

We stratified all patients into simple (<200 cm^3^) and complex (≥200 cm^3^) subgroups based on preoperative uterine volume (Figure 3). Exact sample sizes for each surgical group within the two strata were as follows: MPLH (simple *n* = 28, complex *n* = 90), RASPH (simple *n* = 28, complex *n* = 7), SPLH (simple *n* = 114, complex *n* = 13). Notably, the complex uterine subgroup was severely unbalanced across modalities, with only seven patients in the RASPH arm, limiting the statistical power of intergroup comparisons in this stratum. Adjusted mean operative time, standard error and 95% confidence intervals (CIs) were calculated via a linear interaction model with estimated marginal means, and Bonferroni correction was applied to all within-stratum pairwise comparisons. Global intergroup differences in operative time were significant in both simple and complex subgroups (both *P* < 0.001). Full pairwise comparison outputs including mean differences, standard errors, t statistics, 95% CIs and Bonferroni-adjusted *P*-values are summarized in Table 2.

**TABLE 2:** Pairwise adjusted comparisons of operative time stratified by uterine volume subgroup.

Subgroup	Contrast	Mean difference (min)	SE	*t*-value	95% CI	Bonferroni-adjusted *P*
Simple uterus (<200 cm^3^)	MPLH − RASPH	−58.11	8.23	−7.06	(−74.24, −41.98)	<0.0001
	MPLH − SPLH	−6.48	6.49	−0.998	(−19.19, 6.23)	0.9572
	RASPH − SPLH	51.63	6.49	7.95	(38.92, 64.34)	<0.0001
Complex uterus (≥ 200 cm^3^)	MPLH − RASPH	−54.49	12.1	−4.51	(−78.21, −30.77)	<0.0001
	MPLH − SPLH	−24.31	9.13	−2.66	(−42.20, −6.42)	0.0248
	RASPH − SPLH	30.19	14.4	2.09	(−0.43, 60.81)	0.1122

Adjusted estimates were generated from a linear interaction model with estimated marginal means; Bonferroni correction was implemented for all within-stratum pairwise contrasts. The RASPH complex uterine subgroup only contained seven patients, leading to wide confidence intervals and limited statistical power for intergroup inference.

In the simple small-uterine cohort, RASPH demonstrated significantly longer operative time relative to MPLH and SPLH (both adjusted *P* < 0.001), whereas the difference between MPLH and SPLH did not reach statistical significance (adjusted *P* = 0.9572). In the complex large-uterine cohort, MPLH exhibited significantly shorter operative time than both RASPH (adjusted *P* < 0.0001) and SPLH (adjusted *P* = 0.0248). Although RASPH had a numerically longer mean operative time than SPLH, this intergroup gap failed to achieve statistical significance after Bonferroni correction (adjusted *P* = 0.1122). Notably, the RASPH complex subgroup contained only seven patients, which yielded extremely wide 95% CIs and poor statistical precision. No robust conclusion can be drawn regarding widened intergroup gaps in complex cases due to the severely underpowered RASPH arm.

Nevertheless, this single-volume cutoff could not fully eliminate the substantial baseline imbalance in uterine size across groups. Clinicians tended to assign larger uteri to MPLH in routine practice, leading to non-random case allocation and obvious confounding by indication. All subgroup findings derived from stratified crude comparisons should only be interpreted as exploratory descriptive observations, rather than definitive evidence of inherent differences in surgical efficiency among the three techniques.

## 4 Discussion

This single-center retrospective cohort of 280 patients compared perioperative performance, uterine volume-stratified operative disparities and learning curve profiles across MPLH, RASPH and SPLH. Consistent evidence derived from multivariable regression and IPTW weighting confirmed that robotic single-port hysterectomy carried independent disadvantages in operative efficiency and intraoperative hemorrhage after adjusting for baseline confounding, while MPLH and SPLH exhibited comparable procedural speed with equivalent short-term 30-days safety. Subgroup and learning curve analyses further revealed distinct technical thresholds and interpretive limitations that carry meaningful clinical training and patient-selection implications.

### 4.1 Operative efficiency and safety of minimally invasive hysterectomy techniques

Prior comparative studies have consistently reported longer procedural durations for robotic hysterectomy platforms (5, 8), a trend replicated in our cohort even after excluding robotic docking and pre-setup time to isolate pure console operating efficiency. Two key interpretive limitations should be noted here: first, total incision-to-closure time was recorded for laparoscopic groups while only console duration was captured for robotic cases, creating systematic measurement disparity that may partially widen the observed operative time gap; second, all RASPH procedures were performed by a single surgeon, whereas two separate operators completed MPLH and SPLH cases, potentially introducing unmeasured operator-related confounding despite standardized surgical steps. Clinicians routinely allocated patients with larger uteri to multiport laparoscopy, leading to profound baseline imbalance that distorted unadjusted comparisons. Concordant results from multivariable regression and IPTW weighting confirmed RASPH was independently associated with prolonged operative duration relative to MPLH, while comparable efficiency was observed between MPLH and SPLH.

The discrepancy between unadjusted and adjusted intraoperative blood loss values underscores substantial allocation bias driven by preferential assignment of small-uterus patients to robotic surgery in routine clinical practice. Unadjusted pairwise analyses showed similar hemorrhage levels between MPLH and RASPH, yet SPLH exhibited numerically greater raw blood loss. Following multivariable adjustment for uterine volume, BMI, pelvic adhesions and pathological status, RASPH demonstrated equivalent hemostatic performance to MPLH, whereas SPLH remained independently linked to higher intraoperative blood loss. The increased bleeding risk of SPLH can be explained by instrument crowding within the single-port cavity, which impedes rapid dissection and timely hemostasis during suturing. Even with narrow single-channel spatial constraints and limited institutional robotic experience, RASPH showed no inherent bleeding disadvantage after balancing baseline patient characteristics.

No significant intergroup differences were identified in postoperative hospital stay or POD1 VAS pain scores, and the overall 30-days complication rate remained low and balanced across the three modalities. Identical standardized multimodal analgesia protocols were administered to all participants to minimize confounding of pain assessments; however, only single-day pain recordings were available, lacking serial postoperative pain trajectories for comprehensive comparison. Collectively, MPLH, SPLH and RASPH had comparable short-term perioperative safety profiles, and the operative time disadvantage of RASPH was not caused by robotic docking or system preparation workflows.

### 4.2 Subgroup heterogeneity based on uterine volume

Stratification by a 200 cm^3^ uterine volume cutoff aimed to disentangle technical performance across low- and high-difficulty cases, yet this approach was constrained by severe sample imbalance in the complex uterine subgroup. The tiny RASPH sample within large uterus strata drastically reduced statistical power; while a numerical operative time gap existed between RASPH and SPLH for complex cases, this difference failed to reach statistical significance following Bonferroni correction and cannot be interpreted as definitive technical divergence.

Notably, neither single-variable stratification nor IPTW weighting fully resolved severe imbalance across multiple core baseline covariates (uterine volume, BMI, pelvic adhesion, and tumor pathology) rooted in real-world clinical case assignment, instead of merely uterine size disparity. All subgroup trends observed herein remain exploratory descriptive patterns rather than definitive causal evidence for differences in surgical performance across modalities. Clinicians should exercise caution when extrapolating stratified results to routine practice, as persistent multi-dimensional indication bias limits robust causal inference regarding modality-specific performance in enlarged uteri.

### 4.3 Learning curve characteristics

Cumulative sum-derived learning thresholds offer preliminary training references for surgeons with pre-existing multiport laparoscopic proficiency, though these findings cannot be generalized to surgical novices without foundational minimally invasive experience. MPLH achieved stable operative proficiency at the earliest cumulative case volume, attributable to its standardized, widely adopted procedural framework with fewer intra-cavity instrument coordination demands. SPLH reached proficiency slightly later but maintained a relatively steep learning curve facilitated by modern multi-channel single-port devices and unified surgical steps.

Robot-assisted single-port total hysterectomy required the largest number of consecutive cases to attain consistent operative speed, attributable to unique robotic learning barriers including remote console manipulation, three-dimensional spatial adaptation and coordinated multi-jointed instrument control. Further interpretive caution is warranted for the robotic learning curve: the inflection point occurred at an early stage within only 35 total RASPH cases, leaving a limited number of post-proficiency procedures to benchmark stable performance. Observed robotic learning thresholds therefore require validation through larger prospective robotic cohorts before widespread training guideline implementation.

For surgeons already competent in conventional laparoscopy, a staged training pathway prioritizing single-port laparoscopic mastery before robotic single-port training may shorten robotic learning cycles. This tiered training proposal remains exploratory and requires further prospective validation across multi-surgeon cohorts.

### 4.4 Clinical implications

For routine management of benign and low-risk premalignant uterine lesions, MPLH and SPLH represent first-line minimally invasive choices given balanced operative efficiency and shorter training trajectories. Consistent with our adjusted uterine-volume subgroup analysis showing equivalent operative time between SPLH and MPLH in simple cases (<200 cm^3^), SPLH is particularly suitable for patients with small uteri who prioritize cosmetic outcomes. Though RASPH provides high-fidelity 3D pelvic visualization beneficial for complex anatomical dissection, its intrinsic disadvantages in console operating time and intraoperative hemostasis should be acknowledged during preoperative shared decision-making.

Individual surgical selection ought to integrate uterine size, patient cosmetic preferences, bleeding risk and institutional surgeon experience rather than relying solely on unadjusted crude perioperative metrics. The efficiency limitations of robotic single-port hysterectomy identified in this cohort provide objective real-world evidence to guide personalized surgical planning and structured gynecologic minimally invasive training programs.

### 4.5 Limitations and future directions

This single-center retrospective cohort was subject to inherent selection bias stemming from profound imbalance across four core baseline confounders: preoperative uterine volume, BMI, pelvic adhesion status, and tumor pathological type. Neither multivariable regression nor IPTW weighting could resolve this multi-dimensional imbalance, with negligible improvement in covariate overlap after propensity-score adjustment; stratification by a 200 cm^3^ uterine cutoff also failed to eliminate residual indication bias. Second, surgeon-specific confounding could not be ruled out. Two overlapping surgeons performed MPLH and SPLH, whereas all RASPH operations were completed by one dedicated operator. Although standardized surgical steps reduced inter-operator variation, we could not include surgeon identity as a covariate due to the limited RASPH sample size, which would lead to model overfitting. Third, subgroup analyses for complex uteri (≥200 cm^3^) suffered severe sample imbalance: only seven RASPH patients compared with 90 MPLH and 13 SPLH participants, resulting in low statistical power; all complex-stratum findings remain exploratory rather than definitive. Fourth, only four early malignant cases were enrolled and unevenly distributed among groups, potentially introducing mild residual confounding linked to histotype differences. Fifth, our electronic medical records only captured robotic console time without separate documentation of docking and system setup duration, precluding quantitative differentiation between preparation delays and actual intracavity operating time. Sixth, pain evaluation was limited to a single POD1 VAS measurement. While uniform multimodal analgesia was administered to all participants to minimize confounding, single-point scoring cannot reflect dynamic postoperative pain trajectories and restricts comprehensive intergroup pain comparisons. Seventh, uneven sample sizes and variable baseline laparoscopic experience across surgeons limit the generalizability of both perioperative outcome and learning curve results; we lacked long-term oncologic surveillance and formal cost-effectiveness analyses. Eighth, the RASPH cohort comprised just 35 consecutive procedures, with merely 12 cases recorded after the CUSUM inflection point, weakening the reliability of its proficiency threshold for novice surgeons without prior laparoscopic training.

Future multi-center prospective studies with balanced uterine volume distribution and standardized surgical experience are necessary to validate our adjusted findings and reduce retrospective allocation bias. Larger multi-surgeon cohorts should be recruited to expand the number of early malignant cases and separate surgeon-specific effects from technical performance. Investigators should prospectively record robotic docking and setup time as distinct endpoints to clarify contributors to prolonged console duration. Serial multi-timepoint VAS assessments should replace single POD1 measurements to fully delineate postoperative pain profiles. Additional RASPH patients with enlarged uteri need to be enrolled to achieve adequate statistical power for subgroup analyses, given the current *n* = 7 robotic complex subgroup only yields hypothesis-generating results. The sparse, uneven distribution of malignant lesions also limits robust intergroup oncologic comparisons, even after multivariable and IPTW adjustment.

## 5 Conclusion

This retrospective cohort of 280 patients undergoing minimally invasive total hysterectomy revealed marked baseline uterine volume imbalance that confounded crude intergroup comparisons of operative time and intraoperative blood loss. After adjustment for uterine volume, BMI, pelvic adhesions and pathological diagnosis via multivariable regression and IPTW weighting, RASPH remained independently associated with prolonged operative duration relative to MPLH, while MPLH and SPLH demonstrated comparable operative efficiency. Crude blood loss values suggested similar hemostasis between MPLH and RASPH; after covariate correction, RASPH remained comparable to MPLH in intraoperative hemorrhage, whereas SPLH exhibited significantly greater adjusted blood loss. All three modalities delivered equivalent favorable short-term safety profiles. The crude time disadvantage of RASPH existed in both small and large uterine subgroups. The RASPH complex subgroup only contained seven patients, and the difference between RASPH and SPLH was non-significant after Bonferroni correction; all stratified observations are exploratory only, and severe baseline size imbalance limits causal interpretation of these subgroup results. CUSUM analysis identified proficiency inflection points at 15 cases for MPLH, 19 cases for SPLH and 23 cases for RASPH. Collectively, MPLH and SPLH serve as first-line minimally invasive choices for routine benign and premalignant uterine lesions, whereas RASPH can be reserved for selected cases requiring high-fidelity 3D pelvic visualization, rather than for presumed hemostatic benefits.

## Data Availability

The original contributions presented in this study are included in this article/Supplementary material, further inquiries can be directed to the corresponding author.
