# Supplementary material for: Perioperative outcomes and learning curves of minimally invasive hysterectomy: a comparative analysis of MPLH, RASPH, and SPLH
Source: Front Med (Lausanne). 2026 Jul 15;13:1888457. doi: 10.3389/fmed.2026.1888457 (PMC13416260; doi:10.3389/fmed.2026.1888457)
Supplement: Supplementary file 2 [file Table_1.DOCX]

**Supplementary Table 2. Multivariable linear regression analysis for operative time and intraoperative blood loss, adjusted for uterine volume, BMI, pelvic adhesion and pathological malignancy**

Part A: Multivariable linear regression model for operative time

| Covariate | Regression coefficient (β) | Standard Error (SE) | t value | *P* value | 95% Confidence Interval (CI) |
| --- | --- | --- | --- | --- | --- |
| Intercept | 79.19 | 16.819 | 4.708 | <0.001 | 46.078 – 112.301 |
| Surgical modality – RASPH | 61.747 | 7.201 | 8.575 | <0.001 | 47.571 – 75.923 |
| Surgical modality – SPLH | 7.139 | 6.394 | 1.117 | 0.265 | −5.448 – 19.726 |
| Preoperative uterine volume (cm³) | 0.026 | 0.021 | 1.283 | 0.2 | −0.014 – 0.067 |
| Body mass index (BMI, kg/m²) | 0.752 | 0.599 | 1.256 | 0.21 | −0.427 – 1.931 |
| Pelvic adhesion – Yes | −7.740 | 4.707 | −1.644 | 0.101 | −17.006 – 1.526 |
| Pathological diagnosis – Early malignant | −0.435 | 8.986 | −0.048 | 0.961 | −18.126 – 17.257 |

Model statistics: R²=0.251, Adjusted R²=0.235, F=15.266, *P*<0.001, df=6/273, Total observations=280

Part B: Multivariable linear regression model for intraoperative blood loss

| Covariate | Regression coefficient (β) | Standard Error (SE) | t value | *P* value | 95% Confidence Interval (CI) |
| --- | --- | --- | --- | --- | --- |
| Intercept | 138.29 | 13.2 | 10.48 | <0.001 | 112.30 – 164.27 |
| Surgical modality – RASPH | −3.43 | 5.65 | −0.61 | 0.544 | −14.56 – 7.69 |
| Surgical modality – SPLH | 32.42 | 5.02 | 6.46 | <0.001 | 22.55 – 42.30 |
| Preoperative uterine volume (cm³) | −0.01 | 0.02 | −0.49 | 0.623 | −0.04 – 0.02 |
| BMI (kg/m²) | −0.38 | 0.47 | −0.81 | 0.421 | −1.30 – 0.55 |
| Pathological diagnosis – Early malignant | −2.44 | 3.69 | −0.66 | 0.51 | −9.71 – 4.83 |

Model statistics for blood loss: R²=0.363, Adjusted R²=0.349, F=25.96, df=6/273, Total observations = 280
