# Supplementary material for: Perioperative outcomes and learning curves of minimally invasive hysterectomy: a comparative analysis of MPLH, RASPH, and SPLH
Source: Front Med (Lausanne). 2026 Jul 15;13:1888457. doi: 10.3389/fmed.2026.1888457 (PMC13416260; doi:10.3389/fmed.2026.1888457)
Supplement: Supplementary file 3 [file Table_2.DOCX]

**Supplementary Table 3. Covariate balance before and after IPTW weighting, unadjusted nonparametric effect sizes for operative time and intraoperative blood loss, and IPTW-weighted intergroup comparisons of operative time**

Panel A. Covariate balance before and after IPTW weighting

| **Covariate** | **Pairwise comparison** | **SMD (before weighting)** | **SMD (after weighting)** |
| --- | --- | --- | --- |
| Preoperative uterine volume (cm³) | MPLH vs RASPH | 1.83 | 1.84 |
|  | MPLH vs SPLH | 2.21 | 2.22 |
| Body mass index (BMI, kg/m²) | MPLH vs RASPH | 0.565 | 0.569 |
|  | MPLH vs SPLH | 0.614 | 0.617 |
| Pelvic adhesion (Yes/No) | MPLH vs RASPH | 0.661 | 0.667 |
|  | MPLH vs SPLH | 1.13 | 1.14 |
| Pathological diagnosis (Benign/Malignant) | MPLH vs RASPH | 0.343 | 0.348 |
|  | MPLH vs SPLH | 0.434 | 0.435 |

Panel B. Nonparametric effect sizes from unadjusted Wilcoxon rank-sum tests: Operative time

| **Contrast** | **Z statistic** | **Standardized effect size** | **Magnitude interpretation** |
| --- | --- | --- | --- |
| MPLH − RASPH | −10.87 | 0.65 | Large effect |
| MPLH − SPLH | −1.30 | 0.08 | Negligible effect |
| RASPH − SPLH | −9.41 | 0.56 | Large effect |

Panel C. Nonparametric effect sizes from unadjusted Wilcoxon rank-sum tests: Intraoperative blood loss

| **Contrast** | **Z statistic** | **Standardized effect size** | **Magnitude interpretation** |
| --- | --- | --- | --- |
| MPLH − RASPH | −6.20 | 0.37 | Medium effect |
| MPLH − SPLH | −1.15 | 0.07 | Negligible effect |
| RASPH − SPLH | −5.35 | 0.32 | Medium effect |

Panel D. IPTW-weighted parametric pairwise comparisons of operative time

| **Contrast** | **Weighted mean difference (min)** | **Standard Error (SE)** | **t ratio** | **Bonferroni-adjusted P value** |
| --- | --- | --- | --- | --- |
| MPLH − RASPH | −53.40 | 5.99 | −8.918 | <0.0001 |
| MPLH − SPLH | −3.62 | 3.98 | −0.910 | 1 |
| RASPH − SPLH | 49.78 | 5.94 | 8.381 | <0.0001 |

Panel A presents standardized mean differences (SMD) for baseline covariates; SMD > 0.1 indicates meaningful intergroup imbalance. Panels B and C show Z statistics and standardized nonparametric effect sizes derived from Bonferroni-corrected Wilcoxon rank-sum tests for unadjusted operative time and intraoperative blood loss, respectively. Panel D displays parametric linear regression results after IPTW weight trimming (0.01–0.99). Model-based weighted mean comparisons are interpreted separately from unadjusted nonparametric rank-based analyses. Bonferroni correction was applied for all pairwise contrasts, df = 277. Notably, all SMD values remained substantially above the 0.1 balance threshold after IPTW trimming (0.01–0.99), indicating this weighting method failed to resolve severe multi-dimensional baseline imbalance across the three surgical cohorts.
